# Efficacy and Safety of Extracorporeal Membrane Oxygenation in Patients under Mechanical Ventilation with COVID-19 and Severe Acute Respiratory Distress Syndrome: A Health Technology Assessment

**DOI:** 10.3390/ijerph19106080

**Published:** 2022-05-17

**Authors:** Carlos J. Toro-Huamanchumo, Maribel M. Castro-Reyes, Verónica Peralta, Julissa E. Venancio-Huerta, Pablo R. Puescas-Sanchez, Eric R. Peña-Sanchez

**Affiliations:** 1 Health Technology Assessment Unit, Instituto de Evaluación de Tecnologías en Salud e Investigación—IETSI, EsSalud, Lima 15072, Peru; maribel.castro@essalud.gob.pe (M.M.C.-R.); veronica.peralta@essalud.gob.pe (V.P.); julissa.venancioh@pucp.edu.pe (J.E.V.-H.); eric.penas@essalud.gob.pe (E.R.P.-S.); 2 Unidad Para la Generación y Síntesis de Evidencias en Salud, Universidad San Ignacio de Loyola, Lima 15024, Peru; 3 Laboratorio de Bioingeniería, Pontificia Universidad Católica del Perú, Lima 15088, Peru; 4 Unidad Desconcertada Regional Lambayeque, Seguro Integral de Salud, Chiclayo 14008, Peru; ppuescas@sis.gob.pe; 5 School of Medicine, Universidad de San Martín de Porres, Chiclayo 14012, Peru

**Keywords:** SARS-CoV-2, COVID-19, critical care outcomes, extracorporeal membrane oxygenation

## Abstract

We aimed to assess the efficacy and safety of extracorporeal membrane oxygenation (ECMO) in patients under mechanical ventilation with COVID-19 and severe acute respiratory distress syndrome (ARDS). A systematic review of the literature published in PubMed, Cochrane Library and LILACS databases, was performed. A manual search was also conducted using the reference lists of the studies included in the full-text assessment, as well as a grey-literature search on Google. Additionally, websites of state institutions and organizations developing clinical practice guidelines and health technology assessments were reviewed. The ClinicalTrials.gov website was screened along with the websites of the International Clinical Trial Registry Platform and the National Registry of Health Research Projects of the Peruvian National Institute of Health. No restrictions were applied in terms of language, time, or country. A total of 13 documents were assessed, which included 7 clinical practice guidelines, 3 health technology assessments, 1 systematic review, 1 randomized clinical trial, and 1 observational study. A critical appraisal was conducted for each document. After this, we considered that the currently available evidence is insufficient for a conclusion supporting the use of ECMO in patients under mechanical ventilation with severe ARDS associated to COVID-19 in terms of mortality, safety, and quality of life.

## 1. Introduction

As of 30 March 2022, a total of 481,756,671 confirmed cases and 6,127,981 deaths associated with COVID-19 have been reported worldwide [1]. In Peru, up to March 26, a total of 3,544,862 cases and 212,102 deaths have been reported [2]. Notably, during the pandemic, COVID-19 mortality has exceeded 15% [3,4,5], being particularly higher in patients requiring mechanical ventilation (|) or admitted to an intensive care unit (ICU) [4,6].

Acute respiratory distress syndrome (ARDS) associated with COVID-19 is usually a sign of disease severity. Its pathophysiology includes lung physiology alteration, pulmonary edema, endothelial inflammation, vascular thrombosis, and activation of immune cells. Its prevalence has exceeded 30% [7], and it considerably increases the risk of death [3,8]. During the initial stages of the pandemic, a mortality rate of almost 90% was reported in hospitalized patients with this condition [8].

Current management of ARDS secondary to COVID-19 includes mechanical ventilation [9,10,11,12]. The use of extracorporeal membrane oxygenation (ECMO) has been additionally proposed in patients with COVID-19 and severe respiratory failure [13]. However, the outcomes remain contradictory [14,15,16,17]. Furthermore, the complications and mortality reported in the literature for patients with ARDS associated with COVID-19 subject to ECMO were different from those reported for ARDS induced by other viruses [18,19].

Therefore, the purpose of this review was to assess the efficacy and safety of ECMO in patients under mechanical ventilation with COVID-19 and severe ARDS. This paper is an adapted version the Brief Report No. 01-2022: “Efficacy and safety of extracorporeal membrane oxygenation in patients under mechanical ventilation with COVID-19 and severe acute respiratory distress syndrome” by the Health Technology Assessment and Research Institute (IETSI, by its acronym in Spanish), EsSalud, Lima, Peru.

## 2. Materials and Methods

### 2.1. Clinical Question (PICO)

To achieve the purpose of this health technology assessment, a PICO question including efficacy, safety, and quality of life outcomes was developed (Table 1). This PICO question was proposed by the Health Technology Assessment working group from IETSI and validated by a group of methodologists and clinicians specialized in intensive care medicine.

### 2.2. Search of Evidence

A systematic search of the evidence published up to 2 March 2022, was conducted to identify the best available evidence on the efficacy and safety of ECMO in patients under mechanical ventilation with COVID-19 and severe ARDS. The search was conducted using PubMed, Cochrane Library, and LILACS (Latin American and Caribbean Literature on Health Sciences) databases. Table S1 shows the search strategies and databases used, as well as the results obtained.

A manual search was also conducted using the reference lists of the studies included in the full-text assessment, as well as a grey-literature search on Google. In addition, the search included websites of institutions and organizations developing clinical practice guidelines and health technology assessments (Table S2). The database of the International Network of Agencies for Health Technology Assessment (INAHTA) and the Regional Base of Health Technology Assessment Reports of the Americas (BRISA) were also reviewed.

The World Health Organization (WHO), Pan American Health Organization (PAHO), US Centres for Disease Control and Prevention (CDC), European Centre for Disease Prevention and Control (ECDC), US Food and Drug Administration (FDA), European Medicines Agency (EMA), ECRI platform, Ministry of Health of Peru, and the General Directorate of Medicines, Supplies and Drugs (DIGEMID, by its acronym in Spanish) websites were also reviewed. Finally, ongoing or unpublished clinical trials were searched on the following websites: ClinicalTrials.gov, the International Clinical Trial Registry Platform, and the National Registry of Health Research Projects (PRISA, by its acronym in Spanish) of the Peruvian National Institute of Health.

### 2.3. Selection Criteria

No restrictions were applied in terms of language, time, or country. The search initially focused on the identification of clinical practice guidelines, health technology assessments, and phase-3 randomized clinical trials (RCTs) (or systematic reviews of RCTs).

When searching for clinical practice guidelines, we included those that described a systematic process, those with a detailed description of the scientific evidence search and review process, those with a grading system that determined the strength of their recommendations and the levels of evidence, and those with recommendations about the specific population of our PICO question.

In the case of health technology assessments, the study included those that referred to the population and intervention addressed in our PICO question, those describing a systematic process, those having performed a scientific evidence search and review, and those with a final recommendation or decision or at least a critical appraisal of the evidence included.

Finally, regarding primary studies, when no phase-3 RCTs were found, we considered the inclusion of observational studies assessing the PICO question of interest. Because we found one observational study that met our inclusion criteria, we did not consider reports or case series (as they do not have a control group). In addition, ongoing and/or unpublished RCTs answering the PICO question were searched.

### 2.4. Selection Process

**Figure 1:**
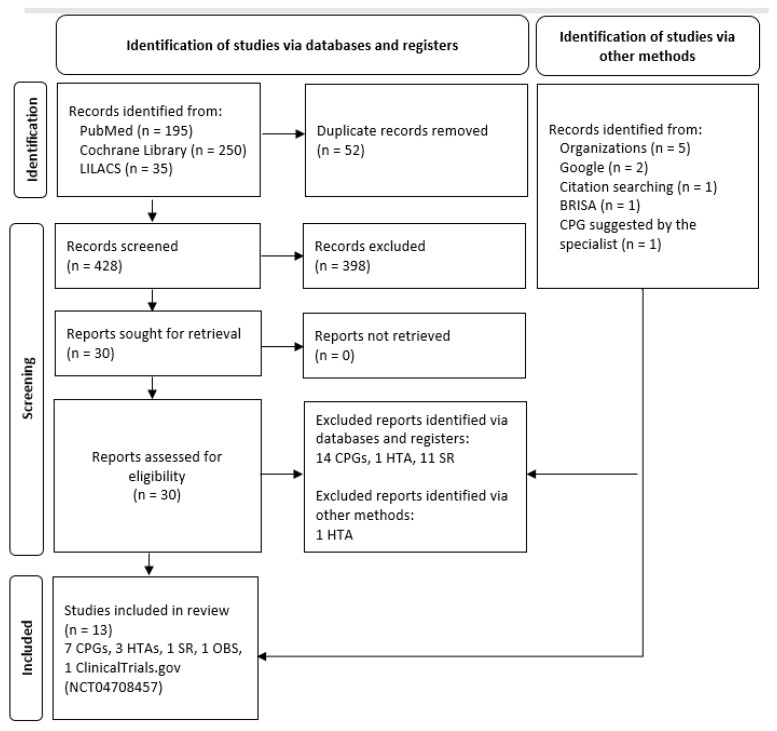
Flowchart of evidence selection. CPG: clinical practice guideline; HTA: health technology assessment; SR: systematic review; OBS: observational study; LILACS: Latin American and Caribbean Health Sciences Literature; BRISA: Regional Database of Health Technology Assessment Reports of the Americas. Flow chart adapted from: Page MJ, et al. The PRISMA 2020 statement: an updated guideline for reporting systematic reviews. BMJ 2021, 372, n71.

Studies were selected using a three-stage framework (Figure 1). The first involved two independent evaluators who reviewed titles and abstracts using the Rayyan web app (https://www.rayyan.ai/). This stage also included the identification of grey-literature documents and allowed for the selection of those studies that should be included and those cases in which more information was necessary. Discrepancies were solved by consensus. The second stage involved one evaluator who conducted a full-text review of the selected studies so as to include those studies fulfilling eligibility criteria. Finally, the third stage consisted of the final selection of the documents to be included in this paper.

## 3. Evidence Description and Critical Appraisal

A total of 13 documents were analysed in this study: 7 clinical practice guidelines, 3 health technology assessments, 1 systematic review (SR), 1 RCT, and 1 observational study (Figure 1). Moreover, 14 clinical practice guidelines, 2 health technology assessments, and 11 SRs that did not answer the PICO question were excluded (Table S3).

### 3.1. Clinical Practice Guidelines

#### 3.1.1. “Living Guidance for Clinical Management of COVID-19: Living Guidance, 23 November 2021”

This clinical practice guideline was developed by the WHO Guideline Development Group (GDG) [20]. The purpose of this document is to make recommendations for the clinical management of COVID-19 patients. Its last update was issued on 23 November 2021.

This clinical practice guideline was developed based on the WHO manual for guidelines development, which provides step-by-step instructions for guideline planning, development, and publication [21]. Moreover, the authors state that their recommendations were based on the Grading of Recommendations Assessment, Development, and Evaluation (GRADE) approach. However, they also reported that the direction and strength of their recommendations were described through symbols (green, red, and yellow) instead of the GRADE formal terminology (strong and conditional recommendations, grading evidence certainty, or good clinical practice points).

Regarding our PICO question, the clinical practice guideline includes a section on “Management of critical COVID-19: acute respiratory distress syndrome (ARDS)”, which issues the following recommendation: “In settings with access to expertise in ECMO, consider referral of patients who have refractory hypoxaemia (e.g., including a ratio of partial pressure of arterial oxygen [PaO_2_] to the fraction of inspired oxygen [FiO_2_] of <50 mmHg for 3 h, a PaO_2_:FiO_2_ of <80 mmHg for >6 h) despite lung-protective ventilation” (this recommendation had a yellow symbol, which suggests that it is a “conditional recommendation in favour of an intervention or recommendation where special care is required in implementation”).

The authors do not provide clear evidence supporting this recommendation, but state that it complies with current international standards for ARDS management, regardless of its cause. They also cite the international guidelines for the management of sepsis and septic shock from the Surviving Sepsis Campaign [22]. Additionally, as a remark for the adult population, the authors of the clinical practice guideline mention that in the EOLIA trial [23], ECMO was associated with a lower risk of the composite outcome of mortality and treatment crossover. Moreover, the findings of a Bayesian posthoc analysis of the same RCT [24] suggested that ECMO most probably reduces mortality. Finally, the clinical practice guideline mentions a cohort of ICU patients with Middle-East Respiratory Syndrome (MERS) [25], suggesting an association between ECMO and a decrease in mortality (compared to conventional therapy). In the case of children, the clinical practice guideline authors state that ECMO could be considered in severe cases, although there is no high-quality evidence about its benefits.

Regarding the methodological limitations of this clinical practice guideline, it should be noted that, even though in some cases the search strategy and evidence selection criteria to answer some of the PICO questions posed by the GDG members were described, this was not the case for the question of interest of our review. Moreover, information about the strengths and limitations of the evidence used is not provided. Owing to the fact that it is unclear whether or not the included literature is pertinent, the reliability of these recommendations decreases.

On the other hand, although the authors state that referral of patients with refractory hypoxemia in spite of lung-protective ventilation should be considered if there is access to ECMO-experienced centres, none of the documents mentioned above [22,23,24,25] provide an answer to our PICO question. This is because none of the studies compares—or includes evidence that compares—the use of ECMO and mechanical ventilation in patients with COVID-19 and severe ARDS. However, because some primary studies are part of the evidence included in some of the clinical practice guidelines mentioned in this paper, we will describe them briefly:

##### EOLIA Trial

The purpose of the EOLIA trial [23] was to determine the effect of early ECMO initiation in patients with the most severe forms of ARDS. Patients assigned to the ECMO group underwent percutaneous venovenous cannulation. Anticoagulation was performed with unfractionated heparin adjusted to a target activated partial thromboplastin time of 40–55 s, or an anti-Xa activity of 0.2–0.3 IU per millilitre. On the other hand, patients in the control group received MECHANICAL VENTILATION pursuant to the “increased recruitment strategy” of the ExPress trial. The use of neuromuscular blocking agents and prolonged periods in a prone position were strongly recommended. Alveolar recruitment maneuvers, inhaled nitric oxide, inhaled prostacyclin, or intravenous almitrine could be used if the oxygenation target values were not achieved. ECMO was allowed in control patients if they showed refractory hypoxemia and if the treating physician considered they did not have irreversible multiorgan failure, and that the ECMO could change their outcome.

The primary outcome was mortality after 60 days. The secondary outcome was treatment failure, which was defined as a crossover to ECMO or death in control patients (composite outcome). No significant differences were observed in mortality after 60 days (44 patients [35%] in the ECMO group vs. 57 [46%] in the control group; relative risk [RR]: 0.76; 95% confidence interval [95% CI]: 0.55–1.04; *p* = 0.09). The authors did find significant differences in the secondary outcome and reported an RR of 0.62 (95% CI: 0.47–0.82; *p* < 0.001).

Notably, the study was interrupted early considering that no significant intergroup differences were observed in terms of mortality after 60 days (primary outcome). The results of the secondary outcome (which are part of the evidence considered by the authors of the WHO clinical practice guideline), should be taken with caution. Firstly, the composite outcome was only considered for the control group and included death and crossover to ECMO, whereas death was the only outcome for the ECMO group. This naturally increases the proportion only in the control group (without ECMO), which results in a possible overestimation of the effect. Secondly, the use of composite outcomes is usually supported by the assumption that the effect on each component will be similar. However, even though death and crossover to ECMO are severity indicators, the RCT authors state that in order to initiate ECMO, the patient had to fulfill the refractory hypoxia criteria, and the opinion of the treating physician should be considered. This difference between the assessment criteria of both components—one being more objective than the other—may be a source of bias and affect global-effect estimations [26].

##### Bayesian Post-Hoc Analysis of the EOLIA Trial

The other study included in the WHO clinical practice guideline was the one by Goligher EC et al. (2018), which consisted of a Bayesian posthoc analysis of the EOLIA trial and concluded that, in the context of different assumptions, early ECMO initiation can reduce mortality compared to conventional lung-protective ventilation [24]. However, different aspects should be considered. First, this post-hoc analysis was not planned, so its findings should be read with caution and only considered as hypothesis generators owing to a possible confirmation bias. Second, repetition of hypothesis testing using different analyses, especially if unplanned, is a possible source of “p-hacking,” increasing the possibility of type 1 errors. Third, the “priors” considered, except for the minimally informative one, were highly flexible. This can result in biased estimations of the treatment effect, which could account for the benefit observed for the intervention. Fourth, the limitations of the analysis, in general, include those inherent to the EOLIA trial, mentioned above.

##### Case Series of Critically Ill Patients with MERS

The last study included in the WHO clinical practice guideline was the one by Arabi YM et al. (2014). The clinical practice guideline authors stated that: “In patients with MERS, ECMO vs. conventional treatment was associated with reduced mortality in a cohort study”. However, when reviewing the study, its authors state that no patient was treated with ECMO, given the presence of multiple comorbidities, thrombocytopenia, and pulmonary affectation [25].

##### Final Comments for This Clinical Practice Guideline

For all the reasons described above—which include the limitations of each study, and the fact that none of them compared ECMO vs. mechanical ventilation use in patients with COVID-19 and severe ARDS—we could conclude that no explicit association was found between the recommendations and evidence cited in this clinical practice guideline.

#### 3.1.2. “Guidelines for Care of Critically Ill Adult Patients with COVID-19 in the Americas. Version 3”

This clinical practice guideline was developed by a group of PAHO experts [27]. The purpose of this document is to provide recommendations for the care of critically ill adult patients with COVID-19 in the ICU. Its last update was issued in May 2021.

To develop this guideline, the authors used the methods described in the PAHO guidelines “Strengthening national evidence-informed guideline programs” [28] and the WHO manual (mentioned in the previous clinical practice guideline). Furthermore, they described the search strategies used for each PICO question, as well as the study selection criteria. Additionally, they mentioned the tools used to analyse the risk of bias in the documents and stated that the recommendations provided in the clinical practice guideline are based on the evidence available and its quality, which was assessed with the GRADE methodology.

Regarding our PICO question, these clinical practice guideline makes the following recommendation: “When possible, patients should receive extracorporeal membrane oxygenation (ECMO) or be referred to an ECMO centre in the following COVID-19 and ARDS critical cases: (i) patients under mechanical ventilation with refractory hypoxia who do not respond to recommended alternative therapies (ventilation optimization, use of rescue therapies, and mechanical ventilation in a prone position). The following patients should not use ECMO: (i) patients with terminal disease or central nervous system damage, or patients who do not want to be resuscitated or who refuse to use ECMO; (ii) patients with significant comorbidities; (iii) patients aged >65 years; and (iv) patients who have received mechanical ventilation for more than 7 days” (conditional recommendation, very low quality of evidence).

Since this document does not refer to any study supporting this recommendation, but only to WHO recommendations, the strengths and limitations of the evidence used cannot be observed. Moreover, if the authors used the same evidence included in the WHO clinical practice guideline, they would be facing the same limitations mentioned above, since this clinical practice guideline showed no explicit association between recommendations and cited evidence.

#### 3.1.3. “COVID-19 Treatment Guidelines Panel. Coronavirus Disease 2019 (COVID-19) Treatment Guidelines”

This document includes the recommendations of the COVID-19 Treatment Guidelines Panel for the management of COVID-19 patients [29]. Its last update about the use of ECMO was in December 2020.

The methodology for the development of this clinical practice guideline is described thoroughly in the document. It also describes the grading system used to measure the strength of recommendations (A, B, and C) and the levels of the quality of the evidence supporting each recommendation (I, IIa, IIb, and III).

Regarding our PICO question, the clinical practice guideline has a section for the management of critically ill adults with COVID-19. The following is thus stated: “There is insufficient evidence to recommend either for or against the use of extracorporeal membrane oxygenation (ECMO) in adults with COVID-19 and refractory hypoxemia”. The authors say that, even though ECMO has been used as a short-term rescue therapy in patients with ARDS secondary to COVID-19 and refractory hypoxemia, there is no concluding evidence suggesting an improvement in clinical outcomes, regardless the cause of such hypoxemic respiratory failure. Moreover, they state that clinical physicians interested in ECMO use should enrol their patients in clinical trials or registries so as to obtain more informative data.

One limitation of this clinical practice guideline is that the search strategy or the evidence selection process are not described. The authors only state that each working group was in charge of identifying relevant information and scientific literature published and of conducting a systematic and comprehensive review.

#### 3.1.4. “Extracorporeal Membrane Oxygenation for COVID-19: Updated 2021 Guidelines from the Extracorporeal Life Support Organization”

This guideline is the updated recommendations made by the Extracorporeal Life Support Organization (ELSO) regarding the role of ECMO in patients with severe cardiopulmonary failure due to COVID-19 [13].

Regarding our PICO question, the authors made the following recommendations: “(i) VV ECMO may be utilized for patients with COVID-19 and severe respiratory failure with expected outcomes comparable to patients supported with V-V ECMO pre-pandemic; and (ii) Rarely, children can require ECMO support for severe ARDS, myocarditis, or multisystem inflammatory disease in children; ECMO patient selection and management should follow conventional guidelines”.

Some of the limitations of this document include the fact that it does not describe any systematic process for the literature review, nor the selection of the studies included. Evidence is not ranked, and recommendations are not graded. Regarding conflicts of interest, the authors are ELSO members and some of them are related to the companies that market/distribute the device. This document was not subject to external peer review, which could have reduced the risk of bias inherent to these conflicts of interest. For these reasons, the relevance of the recommendations suggested in this document is uncertain.

It is important to mention that this document was the only one that did not meet the selection criteria for clinical practice guidelines. However, it was included because it was suggested by the specialists who participated in the validation of the PICO question.

#### 3.1.5. “Surviving Sepsis Campaign Guidelines on the Management of Adults with Coronavirus Disease 2019 (COVID-19) in the ICU: First Update”

This clinical practice guideline is the updated recommendations made by the Surviving Sepsis Campaign working group for the management of critically ill patients with COVID-19 [30]. According to the authors, the methodology used is similar to the one used in the first version [31]. Moreover, they used the GRADE system to assess the quality of evidence and the Evidence to Decision (EtD) framework to develop their recommendations.

Regarding our PICO question, this clinical practice guideline includes a section related to ventilatory support with the following recommendation: “In mechanically ventilated adults with COVID-19 and refractory hypoxemia despite optimizing ventilation, use of rescue therapies, and prone positioning, we suggest using venovenous ECMO, if available, or referring the patient to an ECMO center” (weak recommendation, low quality of evidence).

Two RCTs were identified in the evidence profile of this recommendation, one is EOLIA [23], which was described and analysed above, and the other one is CESAR. We will describe it briefly:

##### CESAR Trial

This was a multicentre RCT conducted to assess the efficacy and safety of ECMO use in adults with severe but potentially reversible respiratory failure and a Murray score of ≥3, or uncompensated hypercapnia with a pH of <7.2 in spite of an optimum conventional treatment [32]. Reversibility was based on the clinical opinion of one of the three on-duty consultants who specialised in ECMO. Patient inclusion was also considered if they had a Murray score of ≥2.5 in order to accelerate essay admission if the patient’s health worsened. Patients were excluded if they had required high-pressure ventilation (maximum inspiratory pressure >30 cmH_2_O) or if they had had high FiO_2_ (>0.8) values for more than 168 h (7 days), signs of intracranial haemorrhage, any other contraindication to limited heparin therapy or any contraindication to the continuity of the active treatment.

The primary outcome was death or severe disability 6 months after randomisation or before hospital discharge. Severe disability was defined as not being able to get out of bed and wash or dress by themselves. Based on this definition, all patients were severely disabled at the time of randomisation, but none of them was disabled before getting the disease and entering the study.

Between July 2001 and August 2006, consultations about 766 potentially eligible patients were performed in 148 centres, 180 of which were enrolled from 68 centres and randomly assigned to a group as follows: 90 to the ECMO group and 90 to the group who received conventional treatment (control). However, only 68 of the patients in the ECMO group (76%) actually ended up with ECMO; three died before transportation, two died during transportation, and 17 received conventional treatment.

An intention-to-treat analysis (ITT) was considered. Consequently, in order to estimate percentages in the ECMO group, the 90 patients randomly assigned to this intervention were still considered as the denominator, even though not all of them had received ECMO. Therefore, 37% (33/90) of ECMO patients had a death or severe disability outcome after 6 months, as opposed to 53% (46/87; this denominator was considered to exclude those with no data about severe disability) (RR 0.69; 95% CI 0.05–0.97; *p* = 0.03). This estimation was made based on 177 patients with known primary outcomes. However, assuming that the three patients in the conventional therapy group without information about disability had all been severely disabled or not severely disabled, the RR would be 0.67 (95% CI: 0.48–0.94; *p* = 0.017) and 0.72 (95% CI: 0.51–1.01; *p* = 0.051), respectively).

A critical analysis of this study suggests: (i) ECMO use is not compared with mechanical ventilation use in patients with COVID-19 and severe ARDS; (ii) Even though the study design is a pragmatic study, which would reflect a “realistic” practice, lack of ECMO use in the treatment arm should be highlighted. Additionally, 22 patients randomly assigned to the ECMO group did not receive this intervention; three of them died before transportation and two during transportation. The 17 remaining patients received conventional treatment and mortality in this group reached 18%. Therefore, the real comparison would be between conventional treatment and referral to a centre which could provide ECMO therapy; (iii) The primary outcome was a composite outcome, so it should be read carefully given the risks mentioned before; (iv) Even though the ITT analysis should be used to avoid effect overestimation, the study only had complete follow-up information for 58% and 36% of ECMO patients and conventional ventilation patients, respectively. For this reason, the results of the analysis should also be read carefully, since the ITT analysis does not decrease the bias introduced by follow-up losses, and when a study has significant follow-up losses, inferences tend to be weak [33].

For all the reasons described above (which include the limitations of each study, and the fact that none of them compared ECMO vs. mechanical ventilation use in patients with COVID-19 and severe ARDS), we could conclude that no explicit association was found between the recommendations and evidence cited in these clinical practice guidelines.

##### Final Comments for This Clinical Practice Guideline

For all the reasons described above—which include the limitations of each study, and the fact that none of them compared ECMO vs. mechanical ventilation use in patients with COVID-19 and severe ARDS—we could conclude that no explicit association was found between recommendations and evidence cited in this clinical practice guideline.

#### 3.1.6. “Australian Guidelines for the Clinical Care of People with COVID-19, v51”

These are the recommendations made by the National COVID-19 Clinical Evidence Taskforce for the clinical care and management of patients with suspected or confirmed COVID-19 [34].

The methodology used to develop these guidelines is described in a technical report [35] and in an additional document stating the evidence search methods [36]. Moreover, the authors state that the clinical practice guideline was developed pursuant to the procedures and requirements of the NHMRC standards for clinical practice guidelines [37], including the use of the GRADE approach to assess evidence certainty and the formulation of recommendations.

In terms of our PICO question, the clinical practice guideline includes sections on ventilatory support in adults and children. Some of its recommendations include “(i) Consider early referral to an ECMO centre for patients developing refractory respiratory failure in mechanically ventilated adults with COVID-19 (despite optimising ventilation, including proning and neuromuscular blockers) (conditional recommendation, very low evidence certainty); (ii) Consider referral to an ECMO centre for venovenous ECMO in mechanically ventilated pregnant women with COVID-19 and refractory respiratory failure (despite optimising ventilation, including prone positioning). Delivery of the baby prior to ECMO to enhance maternal resuscitation should be considered on a case-by-case basis (consensus-based recommendation); and (iii) Consider early referral to an ECMO centre for venovenous or venoarterial ECMO in mechanically ventilated neonates, children and adolescents with COVID-19 with refractory respiratory or cardiovascular failure despite optimising other critical care interventions” (consensus-based recommendation).

The authors report that the evidence supporting the first recommendation was collected from two non-comparative observational studies with critically ill COVID-19 patients subject to ECMO. One study included 1035 patients [15], and the other included 83 [38]. Different aspects of the evidence selected should be considered for this recommendation. First, both studies were conducted in centres highly specialized in the use of ECMO, so the results have limited external validity. Second, they are retrospective studies, so they can suffer selection and information bias, which further limits the extrapolation of results. Third, the studies had incomplete follow-up information about the mortality outcomes after 90 days and complications, so their proportions may be underestimated. Fourth, they are single-arm studies, so no cause-effect conclusions can be made. Fifth, both studies were conducted during the early stages of the pandemic (first semester of 2020). This is relevant owing to the fact that: (i) the mortality after 90 days reported by Barbaro et al. (2020) [15] and Schmidt et al. (2020) [38] was 38% and 36%, respectively. This result is consistent with the pooled global mortality (PGM) reported in a meta-analysis of studies conducted over the same period which included patients with ARDS associated with COVID-19 under corticotherapy (PGM: 39% [95% CI: 23–56%]) [39]; thus, whether ECMO use has additional benefits is even more unclear; (ii) Some of the patients received therapies which are not currently recommended in national and international guidelines, such as hydroxychloroquine. Moreover, recommendations on the management of critically ill COVID-19 patients have undergone further standardization.

Finally, the authors report a lack of studies supporting recommendations for pregnant women and paediatric populations.

#### 3.1.7. “Chemoprophylaxis, Diagnosis, Treatments, and Discharge Management of COVID-19: An Evidence-Based Clinical Practice Guideline (Updated Version)”

This clinical practice guideline includes the recommendations made by the expert group of the Evidence-Based Medicine Chapter of China International Exchange and Promotive Association for Medical and Health Care (CPAM) and the Chinese Research Hospital Association (CRHA) [40].

The methodology for the development of the clinical practice guideline is described thoroughly in the document. The authors used the GRADE system to assess the quality of evidence and the EtD framework to develop their recommendations.

Regarding our PICO question, this clinical practice guideline makes the following recommendation: “ECMO is recommended to treat patients with critical COVID-19, and close monitoring of patient’s vital signs is necessary during use. ECMO should be used in the following situations: (1) early stage (such as severe type with a course of less than 7 days) of critical patients with reversible condition; (2) severe hypoxemia: when using optimized PEEP, PaO_2_/FiO_2_ <100 mmHg after using neuromuscular blocker and prone ventilation; (3) excessive compensatory respiratory acidosis (pH < 7.15) when using optimized mechanical ventilation; (4) excessive inspiratory stress (plateau pressure >30 cmH_2_O) when using lung protective ventilation; (5) using optimized mechanical ventilation setting, the mechanical power is ≥27 J/min; (6) using the optimized mechanical ventilation setting, there is right heart dysfunction due to acute pulmonary heart disease” (recommendation: Grade 1C: strong recommendation, low or very low quality evidence).

It is worth mentioning that this clinical practice guideline does not report the evidence supporting this recommendation, but states that ECMO use is indicated based on “expert evidence.” This implies the following: (i) the presence of uncertainty regarding the guideline recommendations, and (ii) lack of consistency between the quality of evidence and the recommendation.

### 3.2. Health Technology Assessments

#### 3.2.1. “Oxigenação por Membrana Extracorpórea (ECMO) Para Tratamento de Pacientes com COVID-19”

Briefly, this health technology assessment was conducted by the Department of Technology Management and Incorporation and Health Innovation of the Brazilian Ministry of Health [41]. Its purpose was to analyse the scientific evidence on the efficacy and safety of ECMO for the treatment of critically ill COVID-19 patients.

Regarding the efficacy, the results of the studies analysed suggested a higher mortality rate in patients subject to ECMO compared to conventional therapy. However, no significant differences between both groups were observed in the analyses. In addition, the fact that organizations such as WHO, ELSO, and the American Association of Respiratory Care issued recommendations for the use of ECMO resulted in the health technology assessment authors concluding that carefully selected patients with severe ARDS who do not benefit from conventional therapy can obtain good results with venovenous ECMO.

#### 3.2.2. “Membrana de Oxigenación Extracorpórea en Infección por COVID-19. Documentos de Evaluación de Tecnologías Sanitarias, Informe de Respuesta Rápida N° 783”

This report was developed by the IECS Department of Health Technology Assessment. Its purpose was to analyse available evidence on the efficacy and safety of ECMO use in the treatment of COVID-19 infection [42].

Compiled documents included six observational studies (mainly case series) and twelve clinical practice guidelines or recommendation documents. In general, authors stated that the available evidence suggests that ECMO use could be associated to a decrease in the mortality rate of patients with COVID-19 and severe respiratory failure; however, the quality of evidence was extremely poor. For this reason, as of the date of the elaboration of this document, the uncertainty about the efficacy and safety of ECMO use in the treatment of COVID-19 was considered as very high, owing to the fact that available evidence was of extremely low quality and no strong conclusions could be drawn.

#### 3.2.3. “Extracorporeal Membrane Oxygenation (ECMO) for COVID-19 Patients with ARDS”

This health technology assessment was developed by the Health Technology Assessment Unit of the Department of Health of the Republic of the Philippines. Its purpose was to review the current use of ECMO in COVID-19, its efficacy and safety in patients with COVID-19 and ARDS, and possible implications in terms of its implementation resources [43].

The list of compiled documents related to ECMO use consisted of nine clinical practice guidelines or recommendation documents, one health technology assessment, and three full studies (two studies and a case series). However, the authors report that one of the systematic reviews did not include COVID-19 patients and included extracorporeal carbon dioxide removal systems [44]; the systematic review conducted by Haiduc et al. (2020) did not specify the control group in the studies included [45], and the study by Mustafa et al. (2020) did not have any control group [17].

After reviewing the evidence, the health technology assessment authors concluded that, up to that date, the evidence supporting ECMO use in COVID-19 patients was insufficient in terms of mortality rates, hospital stay, and/or symptom resolution. Moreover, they found that ECMO use may increase the risk of haemorrhage, although this was supported through indirect evidence (studies about ARDS before the COVID-19 pandemic). Finally, they also suggested that ECMO use requires several resources in terms of staff, equipment, infrastructure, supplies, and training and maintenance costs.

### 3.3. Systematic Reviews

#### “Extracorporeal Membrane Oxygenation (ECMO) in Patients with Severe COVID-19 Adult Respiratory Distress Syndrome: A Systematic Review and Meta-Analysis”

The purpose of this SR was to estimate the effect of ECMO on the mortality of patients with respiratory failure associated with COVID-19 by means of a systematic review and a meta-analysis [46].

For the outcome of mortality, the authors included different forest plots and pooled analyses. One of them consisted of four studies [47,48,49,50], which also included patients with mechanical ventilation. In the meta-analysis of these studies, the authors found 87.5% and 69.2% mortality rates in ECMO and mechanical ventilation patients, respectively, with a pooled odds ratio (OR) in favour of mechanical ventilation. However, no significant differences were identified (OR: 0.273; 95% CI: 0.07–1.11; *p* = 0.573; I^2^ = 0).

In spite of this, the pooled OR was 0.27, which implies that the mechanical ventilation group had a 73% decrease in the odds of death. This is important since clinical relevance is not the same as statistical significance, and this lack of significance may be attributable to the limited sample size of the studies included. However, it is important to mention that none of the four studies aimed to compare ECMO and mechanical ventilation use in terms of mortality or adverse events, and none of them described patients’ prior therapies or baseline characteristics according to the type of intervention. Moreover, its design introduces a selection bias, limited internal validity, and lack of casual inferences, which is why the findings of this meta-analysis should be interpreted with caution.

### 3.4. Observational Studies

#### 3.4.1. “Extracorporeal Membrane Oxygenation for Coronavirus Disease 2019-Induced Acute Respiratory Distress Syndrome: A Multicenter Descriptive Study”

##### Background of the Study

This is a retrospective observational study conducted in two Chinese hospitals, which included ICU patients with moderate to severe ARDS under mechanical ventilation. Its purpose was to describe the clinical characteristics, the variables associated with ECMO use and the outcomes of patients who received ECMO support for ARDS secondary to COVID-19 [50].

Data was collected from clinical records and included demographic data, medical history, underlying comorbidities, laboratory findings, imaging studies, vital signs, medication, need for continuous kidney replacement therapy, ventilation parameters, and data associated with ECMO. Pneumonia secondary to SARS-CoV-2 was confirmed through imaging and a positive RT-PCR result, whereas ARDS was confirmed as per the Berlin criteria.

The researchers reported that all the participants had followed a standardized and staggered protocol of respiratory support and oxygen therapy. Therefore, ECMO was indicated when lung-protective and prone position strategies were not effective, and when one of the following criteria was present: (1) PaO_2_/FiO_2_ < 50 mmHg for 3 h; (2) PaO_2_/FiO_2_ <80 mmHg for 6 h; (3) arterial pH <7.25 and PaCO_2_ >60 mmHg for 6 h, and a respiratory rate of >35 breaths per minute (rpm); (4) respiratory rate >35 rpm, arterial pH <7.2, and plateau pressure >30 cm H_2_O; and (5) complicated status with cardiogenic shock or cardiac arrest. 

The same strategy was applied to all patients admitted for ECMO: lung-protective ventilation and blood and oxygen flow adjustment based on pulse saturation and arterial gasometry, to maintain the PaO_2_ at 60–80 mmHg and PaCO_2_ at 35–45 mmHg. Moreover, patients received continuous and endovenous heparin infusion based on their activated coagulation time (ACT) and activated partial thromboplastin time (APTT). Target ACT was 160–299 s, whereas target APTT was not higher than two times the upper limit of normal. In patients with a high risk of haemorrhage, the target ACT was 130–160 s, and blood product transfusion was performed if necessary.

##### Results of the Study

From 8 January to 31 March 2020, 129 critically ill patients were admitted to the ICU with pneumonia secondary to SARS-CoV-2. Fifty-nine of them received mechanical ventilation and were eventually included in the study. Their mean age was 65.5 years (56.75–76.0), and 40 of them (67.8%) were male. In the mechanical ventilation cohort, 21 (35.6%) patients underwent ECMO therapy. No significant differences were found between both treatment groups (mechanical ventilation versus mechanical ventilation + ECMO) regarding their baseline characteristics.

In terms of mortality, 24 (63.2%) patients of the mechanical ventilation group died versus 12 (57.1%) patients of the mechanical ventilation + ECMO group (*p* = 0.782). One patient of the mechanical ventilation + ECMO group had bradycardia, which resulted in cardiac arrest. Six patients died due to persistent worsening of lung consolidation which could not be reverted and contracted secondary lung infections by bacteria with multiple resistance to drugs. Three patients died because of septic shock following uncontrolled blood flow infection by *Acinetobacter baumannii* resistant to several drugs. One patient had a cardiac arrest before ECMO initiation and suffered brain death, and another patient had brain haemorrhage and died. General complications in the ECMO group included: bradycardia (*n* = 5), right or left ventricle enlargement and low contractility (*n* = 3) and haemorrhages (*n* = 3). In addition, nine patients received vasopressors and eight developed acute kidney injury and required continuous kidney replacement therapy.

Quality of life was not assessed by the authors.

##### Critical Appraisal of the Study

There are some limitations which are inherent to the study design, such as lack of randomization, which introduces a possible selection bias and affects its internal validity. Sample size or statistical power were not estimated a priori, also introducing the possibility of type II error.

On the other hand, the mean follow-up time is not stated, and the general duration of the study was short, so there is a possible immortal time bias. This is particularly relevant given that, even though significant differences were not observed in the mortality outcome, the actual proportion of dead patients in the ECMO group may be higher, since more than half of the patients died in the course of the study, which was short. This also applies to the complications observed. Furthermore, the authors did not report frequencies or complication types for the mechanical ventilation group, so we could not determine the presence of significant differences between both groups in terms of this outcome.

### 3.5. Randomized Ongoing or Unpublished Clinical Trials

#### The REDEEM Pilot Study: A Feasibility RCT of Early ECMO in Severe Acute Respiratory Infection, including COVID-19, WHO (REDEEM) (NCT04708457)

This pilot study is being conducted in order to determine if early ECMO therapy use in patients with severe acute respiratory infection can be studied by a phase-3 RCT. The study intends to recruit twelve participants between 18 and 70 years of age who have received mechanical ventilation for at least 5 days, with moderate to severe respiratory failure and pneumonitis secondary to severe acute respiratory infection confirmed by laboratory results.

According to ClinicalTrials.gov, this study will be successful if it succeeds in recruiting adult patients, if it shows a difference in favour of early ECMO use, and if it has no safety issues. Approximate end-of-study date: January 2023.

## 4. Discussion

### 4.1. Main Findings

The purpose of this paper was to assess the best available evidence about the efficacy and safety of ECMO in patients under mechanical ventilation with COVID-19 and severe ARDS.

By means of a systematic literature search, seven clinical practice guidelines [13,20,27,29,30,34,40], three health technology assessments [41,42,43], one SR [46], one observational study [50] and one ongoing clinical trial (NCT04708457) were found.

### 4.2. About the Clinical Practice Guidelines

A summary of the recommendations of each guideline is presented in the Table S4. Most clinical practice guidelines, except for NICE, included a recommendation (weak or consensus-based) for ECMO use in patients with ARDS secondary to COVID-19. However, their limitations make us question the pertinence of such recommendations. This is consistent with the study by Luo et al. (2021), who reported that most clinical practice guidelines related to COVID-19 diagnosis and treatment showed low methodological quality [51]. This could be explained by the fact that, during the first year of the pandemic, most guidelines intended to make quick or interim recommendations, so their low quality could be the result of urgency and limited time. This should be considered given that, even though this paper included updated clinical practice guidelines (when available), the evidence used to support the recommendations regarding ECMO use was not updated, so no changes in the studies cited in the original versions (most of them about other populations, without control group or being case series) were introduced. This was reflected by the lack of updated specific recommendations for ECMO ventilatory support.

### 4.3. About Health Technology Assessments

Regarding Health technology assessments, all of them described current evidence about ECMO use in COVID-19 patients as being limited and of low quality. Moreover, only the Health Technology Assessment Unit of the Philippines [43] conducted a detailed analysis of safety, suggesting that ECMO use may increase the risk of haemorrhage. Still, this statement was supported by indirect evidence from pre-pandemic studies about patients with ARDS, but without COVID-19. In addition, this document also assessed the possible effects on the resource needs, concluding that ECMO use required an important number of staff, equipment, infrastructure, supplies, and training and maintenance costs.

### 4.4. About the Systematic Review and the Observational Study

The only SR and observational study included in this paper did not find benefits associated with ECMO use in the population of interest. On the contrary, the SR evidenced a higher proportion of deaths in the ECMO group [46]. Moreover, significant complications were reported in the observational study for the ECMO group [50], although the frequencies and complication types in the control group (patients with mechanical ventilation) were not stated, so it was impossible to find significant differences between both groups in this outcome. It is important to assess this outcome since it is known that some of the most important ECMO complications include bleeding [52,53,54], thromboembolism [55,56] and neurological damage [57,58]. Therefore, given that literature has reported an even higher occurrence of these complications in COVID-19 patients [59,60,61,62], solid evidence is needed regarding the safety profile of this intervention.

## 5. Conclusions

We used a health technology assessment approach to assess the best currently available evidence that could answer our question of interest. The evidence assessed is insufficient for a conclusion supporting the use of ECMO in patients under mechanical ventilation with severe ARDS associated with COVID-19 in terms of mortality, safety, and quality of life.

Randomized, methodologically robust clinical trials should be conducted in order to provide definite recommendations and opinions regarding the efficacy and safety of ECMO use in patients with severe ARDS associated with COVID-19. However, given that conducting these types of research may be difficult in some contexts, the development of observational, comparative studies could be equally valuable.

## Figures and Tables

**Table 1:** PICO question.

**Population**	Patients under mechanical ventilation, with confirmed COVID-19 * and severe acute respiratory distress syndrome **
**Intervention**	Extracorporeal Membrane Oxygenation
**Control**	Mechanical ventilation
**Outcome**	Efficacy: Mortality, Length of stay (in hospital or ICU), Quality of lifeSafety: Adverse events related to the procedure, Adverse events related to the device

* With positive reverse transcription polymerase chain reaction (RT-PCR) for SARS-CoV-2. ** Severity was defined based on the Berlin criteria: arterial oxygen pressure/fraction of inspired oxygen (PaO_2_/FiO_2_) ≤100 mmHg, with a positive end-expiratory pressure (PEEP) ≥5 cm H_2_O.

## Data Availability

Not applicable.

## Supplementary Materials

The following supporting information can be downloaded at: https://www.mdpi.com/article/10.3390/ijerph19106080/s1, Table S1: Search Strategies; Table S2: List of Institutions and Organizations That Develop or Comprise Clinical Practice Guidelines and Health Technology Assessments Whose Webpages Were Reviewed; Table S3: List of Documents Excluded after Full-Text Revision; Table S4: Summary of Recommendations of Each Clinical Practice Guideline.

## References

[1] World Health Organization (2022). WHO Coronavirus (COVID-19) Dashboard. https://covid19.who.int/.

[2] Ministerio de Salud (2022). Sala Situacional COVID-19 Perú [Internet]. https://covid19.minsa.gob.pe/sala_situacional.asp.

[3] Abate S.M., Checkol Y.A., Mantefardo B. (2021). Global prevalence and determinants of mortality among patients with COVID-19: A systematic review and meta-analysis. Ann. Med. Surg..

[4] Noor F.M., Islam M.M. (2020). Prevalence and Associated Risk Factors of Mortality Among COVID-19 Patients: A Meta-Analysis. J. Community Health.

[5] Dessie Z.G., Zewotir T. (2021). Mortality-related risk factors of COVID-19: A systematic review and meta-analysis of 42 studies and 423,117 patients. BMC Infect. Dis..

[6] Khamis F., Memish Z., Bahrani M.A., Dowaiki S.A., Pandak N., Bolushi Z.A., AlSalmi I., Al-Zakwanie I. (2021). Prevalence and predictors of in-hospital mortality of patients hospitalized with COVID-19 infection. J. Infect. Public Health.

[7] Tzotzos S.J., Fischer B., Fischer H., Zeitlinger M. (2020). Incidence of ARDS and outcomes in hospitalized patients with COVID-19: A global literature survey. Crit. Care.

[8] Hendrickson K.W., Peltan I.D., Brown S.M. (2021). The Epidemiology of Acute Respiratory Distress Syndrome Before and After Coronavirus Disease 2019. Crit. Care Clin..

[9] Sasidharan S., Singh V., Singh J., Madan G.S., Dhillon H.S., Dash P.K., Shibu B., Dhillon G.K. (2021). COVID-19 ARDS: A Multispecialty Assessment of Challenges in Care, Review of Research, and Recommendations. J. Anaesthesiol. Clin. Pharmacol..

[10] Gattinoni L., Gattarello S., Steinberg I., Busana M., Palermo P., Lazzari S., Romitti F., Quintel M., Meissner K., Marini G.G. (2021). COVID-19 pneumonia: Pathophysiology and management. Eur. Respir. Rev..

[11] Kondili E., Makris D., Georgopoulos D., Rovina N., Kotanidou A., Koutsoukou A. (2021). COVID-19 ARDS: Points to Be Considered in Mechanical Ventilation and Weaning. J. Pers. Med..

[12] Mega C., Cavalli I., Ranieri V.M., Tonetti T. (2022). Protective ventilation in patients with acute respiratory distress syndrome related to COVID-19: Always, sometimes or never?. Curr. Opin. Crit. Care.

[13] Badulak J., Antonini M.V., Stead C.M., Shekerdemian L., Raman L., Paden M.L., Agerstrand C., Bartlett R.H., Barrett N., Combes A. (2021). Extracorporeal Membrane Oxygenation for COVID-19: Updated 2021 Guidelines from the Extracorporeal Life Support Organization. ASAIO.

[14] Henry B.M., Lippi G. (2020). Poor survival with extracorporeal membrane oxygenation in acute respiratory distress syndrome (ARDS) due to coronavirus disease 2019 (COVID-19): Pooled analysis of early reports. J. Crit. Care.

[15] Barbaro R.P., MacLaren G., Boonstra P.S., Iwashyna T.J., Slutsky A.S., Fan E., Pham T.T. (2020). Extracorporeal membrane oxygenation support in COVID-19: An international cohort study of the Extracorporeal Life Support Organization registry. Lancet.

[16] Zeng Y., Cai Z., Xianyu Y., Yang B.X., Song T., Yan Q. (2020). Prognosis when using extracorporeal membrane oxygenation (ECMO) for critically ill COVID-19 patients in China: A retrospective case series. Crit. Care.

[17] Mustafa A.K., Alexander P.J., Joshi D.J., Tabachnick D.R., Cross C.A., Pappas P.S., Tatooles A.J. (2020). Extracorporeal Membrane Oxygenation for Patients With COVID-19 in Severe Respiratory Failure. JAMA Surg..

[18] Seeliger B., Doebler M., Hofmaenner D.A., Wendel-Garcia P.D., Schuepbach R.A., Schmidt J.J., Welte T.M.D., Tobias M.D., Hoeper M.M.D., Gillmann H.M.D. (2022). Intracranial Hemorrhages on Extracorporeal Membrane Oxygenation: Differences Between COVID-19 and Other Viral Acute Respiratory Distress Syndrome. Crit. Care Med..

[19] Raff L.A., Reid T.D., Johnson D., Raff E.J., Schneider A.B., Charles A.G., Gallaher J.R. (2022). Comparative outcomes between COVID-19 and influenza patients placed on veno-venous extracorporeal membrane oxygenation for severe ARDS. Am. J. Surg..

[20] World Health Organization (2021). Living Guidance for Clinical Management of COVID-19: Living Guidance.

[21] World Health Organization (2014). WHO Handbook for Guideline Development.

[22] Evans L., Rhodes A., Alhazzani W., Antonelli M., Coopersmith C.M., French C., Levy M. (2021). Surviving Sepsis Campaign: International Guidelines for Management of Sepsis and Septic Shock 2021. Crit. Care Med..

[23] Combes A., Hajage D., Capellier G., Demoule A., Lavoué S., Guervilly C., Alexandre D., Sylvain L., Christophe G., Da Silva D. (2018). Extracorporeal Membrane Oxygenation for Severe Acute Respiratory Distress Syndrome. N. Engl. J. Med..

[24] Goligher E.C., Tomlinson G., Hajage D., Wijeysundera D., Fan E., Jüni P., Brodie D., Slutsky A.S., Combes A. (2018). Extracorporeal Membrane Oxygenation for Severe Acute Respiratory Distress Syndrome and Posterior Probability of Mortality Benefit in a Post Hoc Bayesian Analysis of a Randomized Clinical Trial. JAMA.

[25] Arabi Y.M., Arifi A.A., Balkhy H.H., Najm H., Aldawood A.S., Ghabashi A., Hawa H., Alothman A., Khaldi A., Al Raiy B. (2014). Clinical course and outcomes of critically ill patients with Middle East respiratory syndrome coronavirus infection. Ann. Intern. Med..

[26] Montori V., Permanyer-Miralda G., Ferreira-González I., Busse J., Pacheco-Huergo V., Bryant D., Alonso J., A Akl E., Domingo-Salvany A., Mills E. (2005). Validity of composite end points in clinical trials. BMJ.

[27] Organización Panamericana de la Salud (2021). Guía Para el Cuidado de Pacientes Adultos Críticos con COVID-19 en las Américas.

[28] Organización Panamericana de la Salud (2018). Directriz para el Fortalecimiento de los Programas Nacionales de GUÍAS Informadas por la Evidencia. Una Herramienta para la Adaptación e Implementación de Guías en las Américas.

[29] National Institutes of Health (2022). Coronavirus Disease 2019 (COVID-19) Treatment Guidelines.

[30] Alhazzani W., Evans L., Alshamsi F., Møller M.H., Ostermann M., Prescott H.C., Arabi Y.M., Loeb M., Gong M.N., Fan E. (2021). Surviving Sepsis Campaign Guidelines on the Management of Adults With Coronavirus Disease 2019 (COVID-19) in the ICU: First Update. Crit. Care Med..

[31] Alhazzani W., Møller M.H., Arabi Y.M., Loeb M., Gong M.N., Fan E., Oczkowski S., Levy M.M., Derde L., Dzierba A. (2020). Surviving Sepsis Campaign: Guidelines on the Management of Critically Ill Adults with Coronavirus Disease 2019 (COVID-19). Crit. Care Med..

[32] Peek G.L., Mugford M., Tiruvoipati R., Wilson A., Allen E., Thalanany M.M., Hibbert C.L., Truesdale A., Clemens F., Cooper N. (2009). Efficacy and economic assessment of conventional ventilatory support versus extracorporeal membrane oxygenation for severe adult respiratory failure (CESAR): A multicentre randomised controlled trial. Lancet.

[33] Montori V.M., Guyatt G.H. (2001). Intention-to-treat principle. Can. Med. Assoc. J..

[34] Australian National COVID-19 Clinical Evidence Taskforce (2022). Australian Guidelines for the Clinical Care of People with COVID-19.

[35] National COVID-19 Clinical Evidence Taskforce (2020). Methods for Living Guidelines for Management and Care of People in Primary, Hospital and Critical Care with Suspected or Confirmed COVID-19 Infection: Technical Report [Internet]. https://covid19evidence.net.au/wp-content/uploads/Technical-Report_20201022.pdf?=201022-72442.

[36] National COVID-19 Clinical Evidence Taskforce (2021). Search Methods Version 3, 1 July 2021 [Internet]. https://covid19evidence.net.au/wp-content/uploads/NC19CET-Search-Methods-V3.pdf.

[37] National Health and Medical Research Council (2016). 2016 NHMRC Standards for Guidelines [Internet]. https://www.nhmrc.gov.au/guidelinesforguidelines/standards.

[38] Schmidt M., Hajage D., Lebreton G., Monsel A., Voiriot G., Levy D., Baron E., Beurton A., Chommeloux J., Meng P. (2020). Extracorporeal membrane oxygenation for severe acute respiratory distress syndrome associated with COVID-19: A retrospective cohort study. Lancet Respir. Med..

[39] Hasan S.S., Capstick T., Ahmed R., Kow C.S., Mazhar F., Merchant H.A., Zaidi S.T.R. (2020). Mortality in COVID-19 patients with acute respiratory distress syndrome and corticosteroids use: A systematic review and meta-analysis. Expert Rev. Respir. Med..

[40] Jin Y.-H., Zhan Q.-Y., Peng Z.-Y., Ren X.-Q., Yin X.-T., Cai L., Yuan Y.-F., Yue J.-R., Zhang X.-C., Yang Q.-W. (2020). Chemoprophylaxis, diagnosis, treatments, and discharge management of COVID-19: An evidence-based clinical practice guideline (updated version). Mil. Med. Res..

[41] Ministério Da Saúde (2020). Oxigenação por Membrana Extracorpórea (ECMO) Para Tratamento de Pacientes com COVID-19.

[42] Instituto de Efectividad Clínica y Sanitaria (2020). Membrana de Oxigenación Extracorpórea en Infección por COVID-19. Documentos de Evaluación de Tecnologías Sanitarias.

[43] Health Technology Assessment Unit, DOH (2020). Extracorporeal Membrane Oxygenation (ECMO) for COVID-19 Patients with ARDS.

[44] Aretha D., Fligou F., Kiekkas P., Karamouzos V., Voyagis G. (2019). Extracorporeal Life Support: The Next Step in Moderate to Severe ARDS-A Review and Meta-Analysis of the Literature. BioMed Res. Int..

[45] Haiduc A.A., Alom S., Melamed N., Harky A. (2020). Role of extracorporeal membrane oxygenation in COVID-19: A systematic review. J. Card. Surg..

[46] Elsayed H.H., Hassaballa A.S., Ahmed T.A., Gumaa M., Sharkawy H.Y. (2021). Extracorporeal membrane oxygenation (ECMO) in patients with severe COVID-19 adult respiratory distress syndrome: A systematic review and meta-analysis. Cardiothorac. Surg..

[47] Ruan Q., Yang K., Wang W., Jiang L., Song J. (2020). Clinical predictors of mortality due to COVID-19 based on an analysis of data of 150 patients from Wuhan, China. Intensive Care Med..

[48] Guan W.J., Ni Z.Y., Hu Y., Liang W.H., Qu C.Q., He J.X., Liu L., Shan H., Lei C.L., Hui D.S.C. (2020). Clinical Characteristics of Coronavirus Disease 2019 in China. N. Engl. J. Med..

[49] Li T., Yin P.-F., Li A., Shen M.R., Yao Y.-X. (2021). Acute Respiratory Distress Syndrome Treated With Awake Extracorporeal Membrane Oxygenation in a Patient With COVID-19 Pneumonia. J. Cardiothorac. Vasc. Anesthesia.

[50] Yang X., Cai S., Luo Y., Zhu F., Hu M., Zhao Y., Zheng R., Li X., Hu B., Peng Z. (2020). Extracorporeal Membrane Oxygenation for Coronavirus Disease 2019-Induced Acute Respiratory Distress Syndrome: A Multicenter Descriptive Study*. Crit. Care Med..

[51] Luo X., Liu Y., Ren M., Zhang X., Janne E., Lv M., Wang Q., Song Y., Mathew J.L., Ahn H.S. (2021). Consistency of recommendations and methodological quality of guidelines for the diagnosis and treatment of COVID-19. J. Evid.-Based Med..

[52] Mazzeffi M., Greenwood J., Tanaka K., Menaker J., Rector R., Herr D., Kon Z., Lee J., Griffith B., Rajagopal K. (2016). Bleeding, Transfusion, and Mortality on Extracorporeal Life Support: ECLS Working Group on Thrombosis and Hemostasis. Ann. Thorac. Surg..

[53] Sklar M.C., Sy E., Lequier L., Fan E., Kanji H.D. (2016). Anticoagulation Practices during Venovenous Extracorporeal Membrane Oxygenation for Respiratory Failure. A Systematic Review. Ann. Am. Thorac. Soc..

[54] Olson S.R., Murphree C.R., Zonies D., Meyer A.D., Mccarty O.J.T., Deloughery T.G., Shatzel J.J. (2020). Thrombosis and Bleeding in Extracorporeal Membrane Oxygenation (ECMO) Without Anticoagulation: A Systematic Review. ASAIO J..

[55] Hartley E.L., Singh N., Barrett N., Wyncoll D., Retter A. (2019). Screening pulmonary angiogram and the effect on anticoagulation strategies in severe respiratory failure patients on venovenous extracorporeal membrane oxygenation. J. Thromb. Haemost..

[56] Parzy G., Daviet F., Persico N., Rambaud R., Scemama U., Adda M., Guervilly C., Hraiech S., Chaumoitre K., Roch A. (2020). Prevalence and Risk Factors for Thrombotic Complications Following Venovenous Extracorporeal Membrane Oxygenation. Crit. Care Med..

[57] Chapman J.T., Breeding J., Kerr S.J., Bajic M., Nair P., Buscher H. (2020). CNS Complications in Adult Patients Treated With Extracorporeal Membrane Oxygenation. Crit. Care Med..

[58] Nasr D.M., A Rabinstein A. (2015). Neurologic Complications of Extracorporeal Membrane Oxygenation. J. Clin. Neurol..

[59] Bemtgen X., Zotzmann V., Benk C., Rilinger J., Steiner K., Asmussen A., Bode C., Wengenmayer T., Maier S., Staudacher D.L. (2020). Thrombotic circuit complications during venovenous extracorporeal membrane oxygenation in COVID-19. J. Thromb. Thrombolysis.

[60] Arachchillage D.J., Rajakaruna I., Scott I., Gaspar M., Odho Z., Banya W., Vlachou A., Isgro G., Cagova L., Wade J. (2021). Impact of major bleeding and thrombosis on 180-day survival in patients with severe COVID-19 supported with veno-venous extracorporeal membrane oxygenation in the United Kingdom: A multicentre observational study. Br. J. Haematol..

[61] Kannapadi N.V., Jami M., Premraj L., Etchill E.W., Giuliano K., Bush E.L., Kim B.S., Seal S., Whitman G., Cho S.-M. (2021). Neurological Complications in COVID-19 Patients With ECMO Support: A Systematic Review and Meta-Analysis. Heart Lung Circ..

[62] Masur J., Freeman C., Mohan S. (2020). A Double-Edged Sword: Neurologic Complications and Mortality in Extracorporeal Membrane Oxygenation Therapy for COVID-19–Related Severe Acute Respiratory Distress Syndrome at a Tertiary Care Center. Am. J. Neuroradiol..

