# Supplementary material for: Efficacy and Safety of Extracorporeal Membrane Oxygenation in Patients under Mechanical Ventilation with COVID-19 and Severe Acute Respiratory Distress Syndrome: A Health Technology Assessment
Source: Int J Environ Res Public Health. 2022 May 17;19(10):6080. doi: 10.3390/ijerph19106080 (PMC9140858; doi:10.3390/ijerph19106080)
Supplement: Supplementary file 1 [file ijerph-19-06080-s001.zip › ijerph-1716023-supplementary.pdf]

Table S1. Search Strategies

Database		Search Strategy	Results
**PubMed**	#1	(Coronavirus[Mesh] OR SARS-CoV-2[Mesh] OR COVID-19[Mesh] OR Corona Virus[tiab] OR COVID-19[tiab] OR COVID19*[tiab] OR 2019-nCoV[tiab] OR SARS-CoV-2[tiab] OR SARS-CoV2[tiab] OR SARSCoV2[tiab] OR (Pneumonia[tiab] AND Wuhan[tiab] AND 2019[tiab]) OR Coronavir*[tiab] OR Coronovir*[tiab] OR Virus Corona[tiab] OR Corono Virus[tiab] OR HCov*[tiab] OR CV19*[tiab] OR CV-19[tiab] OR N-Cov[tiab]) AND (Extracorporeal Membrane Oxygenation[Mesh] OR ECMO*[tiab] OR Membrane Oxygenation[tiab] OR Extracorporeal Membrane[tiab] OR Extracorporeal Life[tiab] OR ECLS[tiab] OR Extracorporeal Gas[tiab] OR Cardiopulmonary Support[tiab] OR Cardiopulmonary Resuscitat*[tiab] OR ECPR[tiab] OR ECLS[tiab] OR Cardiohelp[tiab] OR Capiox[tiab] OR Quadrox[tiab] OR Nautilus[tiab] OR Hilite[tiab]) AND (Systematic Review[sb] OR Systematic Review[tiab] OR Meta-Analysis[pt] OR Meta-Analys*[tiab] OR “Cochrane Database Syst Rev”[ta] OR Metaanalysis[tiab] OR Metanalysis[tiab] OR (MEDLINE[tiab] AND Cochrane[tiab]) OR Guideline[pt] OR Practice Guideline[pt] OR Guideline*[ti] OR Guide Line*[tiab] OR Consensus[tiab] OR Recommendation*[ti] OR Randomized Controlled Trial[pt] OR Random*[ti] OR Controlled Trial*[tiab] OR Control Trial*[tiab] OR Technology Assessment, Biomedical[Mesh] OR Technology Assessment[tiab] OR Technology Appraisal[tiab] OR HTA[tiab] OR Overview[ti] OR (Review[ti] AND Literature[ti]))	195
**LILACS**	#1	(MH Coronavirus OR MH SARS-CoV-2 OR MH COVID-19 OR Corona OR COVID-19 OR COVID19$ OR 2019-nCoV OR SARS-CoV-2 OR SARS-CoV2 OR SARSCoV2 OR Coronavir$ OR Coronovir$ OR Corono OR HCov$ OR CV19$ OR CV-19$ OR N-Cov) AND (MH Extracorporeal Membrane Oxygenation OR ECMO$ OR ((Membran$ OR Gas OR Support OR Soporte) AND (Oxygena$ OR Oxigena$ OR Extracorporea$)) OR ECLS OR ((Cardiopulmonar$ OR Cardio-Pulmonar$) AND (Support OR Soporte OR Resusci$)) OR ECPR OR ECLS OR Cardiohelp OR Capiox OR Quadrox OR Nautilus OR Hilite) [Words]	35
**Cochrane**	#1	MeSH descriptor: [Coronavirus] explode all trees	538
	#2	MeSH descriptor: [SARS-CoV-2] explode all trees	525
	#3	MeSH descriptor: [COVID-19] explode all trees	771
	#4	(Coron* NEAR/1 Virus):ti,ab,kw	272
	#5	COVID-19:ti,ab,kw	7841
	#6	COVID19*:ti,ab,kw	408
	#7	“2019-nCoV”:ti,ab,kw	11
	#8	SARS-CoV-2:ti,ab,kw	315
	#9	SARS-CoV2:ti,ab,kw	73
	#10	SARCoV2:ti,ab,kw	3
	#11	(Pneumonia AND Wuhan AND 2019):ti,ab,kw	102
	#12	Coronavir*:ti,ab,kw	4816
	#13	Coronovir*:ti,ab,kw	1
	#14	HCov*:ti,ab,kw	17
	#15	CV19*:ti,ab,kw	0
	#16	CV-19:ti,ab,kw	10
	#17	N-Cov*:ti,ab,kw	29
	#18	#1 OR #2 OR #3 OR #4 OR #5 OR #6 OR #7 OR #8 OR #9 OR #10 OR #11 OR #12 OR #13 OR #14 OR #15 OR #16 OR #17	8380
	#19	MeSH descriptor: [Extracorporeal Membrane Oxygenation] explode all trees	191
	#20	ECMO*:ti,ab,kw	738
	#21	(Membrane NEAR/3 Oxygenation):ti,ab,kw	745
	#22	(Extracorporeal NEAR/3 Membrane):ti,ab,kw	730
	#23	(Extracorporeal NEAR/3 Life):ti,ab,kw	72

#24	ECLS*:ti,ab,kw	54
#25	(Extracorporeal NEAR/3 Gas):ti,ab,kw	24
#26	(Cardiopulmonary NEAR/3 Support):ti,ab,kw	60
#27	(Cardiopulmonary NEAR/3 Resuscitat*):ti,ab,kw	2382
#28	ECPR:ti,ab,kw	31
#29	ECLS:ti,ab,kw	54
#30	Cardiohelp:ti,ab,kw	1
#31	Capiox:ti,ab,kw	22
#32	Quadrox:ti,ab,kw	9
#33	Nautilus:ti,ab,kw	24
#34	Hilite:ti,ab,kw	3
#35	#19 OR #20 OR #21 OR #22 OR #23 OR #24 OR #25 OR #26 OR #27 OR #28 OR #29 OR #30 OR #31 OR #32 OR #33 OR #34	3559
#36	#18 AND #35	250

Table S2. List of Institutions and Organizations That Develop or Comprise Clinical Practice Guidelines and Health Technology Assessments Whose Webpages Were Reviewed

National Institute for Health and Care Excellence (NICE)
Agency for Healthcare Research and Quality's (AHRQ)
Scottish Intercollegiate Guidelines Network (SIGN)
Guidelines International Network (GIN), Scottish Medicines Consortium (SMC)
National Health and Medical Research Council (NHMRC)
Canadian Agency for Drugs and Technologies in Health (CADTH)
Institut für Qualität und Wirtschaftlichkeit im Gesundheitswesen (IQWiG)
Haute Autorité de Santé (HAS)
Agenzia nazionale per i servizi sanitari regionali (AGENAS)
Centro Nacional de Excelencia Tecnológica en Salud (CENETEC)
Comissão Nacional de Incorporação de Tecnologias no Sistema Único de Saúde (CONITEC)
Instituto de Evaluación Tecnológica en Salud (IETS)
Instituto de Efectividad Clínica y Sanitaria (IECS)

Table S3. List of Documents Excluded after Full-Text Revision

First Author (Year)	Title	Reason for Exclusion
Shekar K (2020)	Extracorporeal Life Support Organization Coronavirus Disease 2019 Interim Guidelines: A Consensus Document from an International Group of Interdisciplinary Extracorporeal Membrane Oxygenation Providers	It is an earlier version of the paper by Badulak J et al. (2021).
Ronco (2021)	Extracorporeal Blood Purification and Organ Support in the Critically Ill Patient during COVID-19 Pandemic: Expert Review and Recommendation	Document is a review article.
Ko HK (2021)	Consensus statement and recommendations on the treatment of COVID-19: 2021 Update	Consensus document.
Mufti HN (2021)	The Saudi Critical Care Society extracorporeal life support chapter guidance on utilization of veno-venous extracorporeal membrane oxygenation in adults with acute respiratory distress syndrome and special considerations in the era of coronavirus disease 2019	Document does not present a system of evidence hierarchy or gradation of its recommendations.

Jeong IS (2021)	Extracorporeal Membrane Oxygenation for Coronavirus Disease 2019: Expert Recommendations from The Korean Society for Thoracic and Cardiovascular Surgery	Document does not present a system of evidence hierarchy or gradation of its recommendations.
Camporota L (2021)	Consensus on the referral and admission of patients with severe respiratory failure to the NHS ECMO service	Document does not present a system of evidence hierarchy or gradation of its recommendations.
Rajagopal K (2021)	Advanced Pulmonary and Cardiac Support of COVID-19 Patients: Emerging Recommendations from ASAIO—A "Living Working Document	Document does not present a system of evidence hierarchy or gradation of its recommendations.
Chinese Society of Extracorporeal Life Support (2020)	Recommendations on extracorporeal life support for critically ill patients with novel coronavirus pneumonia	Document does not present a system of evidence hierarchy or gradation of its recommendations.
Bein B (2020)	SARS CoV-2/COVID-19: Evidence-Based Recommendation on Diagnosis and Therapy	Document does not present a system of evidence hierarchy or gradation of its recommendations.
Chen Z (2020)	Diagnosis and treatment recommendation for pediatric COVID-19 (the second edition)	Document does not present a system of evidence hierarchy or gradation of its recommendations.
Wiedemann D (2020)	Recommendations for extracorporeal membrane oxygenation (ECMO) in COVID-19 patients: Consensus paper of the Medical University of Vienna	Document does not present a system of evidence hierarchy or gradation of its recommendations.
Ñamendys SA (2020)	Recommendations for the management of critically ill adult patients with COVID-19	Document does not present a system of evidence hierarchy or gradation of its recommendations.
Universidad de Antioquia, (2020)	Síntesis Rápida: Tratamiento farmacológico de la infección COVID-19 en adultos. Actualización	Documento no presenta un sistema de jerarquización de la evidencia o gradación de sus recomendaciones.
Kache S (2020)	COVID-19 PICU guidelines: for high- and limited-resource settings	Document does not present a system of evidence hierarchy or gradation of its recommendations.
Comissão Nacional de Incorporação de Tecnologias no Sistema Único de Saúde (2021)	Oxigenação Extracorpórea (ECMO) para Suporte de Pacientes com Insuficiência Respiratória Grave e Refratária	The population considered was patients with ARDS caused by any viral infection. Although they included studies in patients with COVID-19, their recommendations and final decision were not specific to this population of interest in the PICO question of our review.
Canadian Agency for Drugs and Technologies in Health (2021)	CADTH Health Technology Review: Extracorporeal Membrane Oxygenation for Adults and Children With Severe Respiratory Failure	The objective of the study was to conduct a qualitative literature review on the perspectives and experiences of patients, family members, caregivers and health care workers regarding the use of ECMO, not being in accordance with the PICO question of our review.
Haiduc AA (2020)	Role of extracorporeal membrane oxygenation in COVID-19: A systematic review	Document considered, indistinctly, case reports, observational studies without a control group and/or studies that did not correspond to the population of interest in the PICO question of our review.

Hu BS (2020)	Extracorporeal membrane oxygenation (ECMO) in patients with COVID-19: a rapid systematic review of case studies	Document only considered reports and case series.
Kannapadi NV (2021)	Neurological Complications in COVID-19 Patients With ECMO Support: A Systematic Review and Meta-Analysis	Authors only considered studies whose population received ECMO therapy (no control group).
Ramanathan K (2021)	Extracorporeal membrane oxygenation for COVID-19: a systematic review and meta-analysis	Authors only considered studies whose population received ECMO therapy (no control group).
Kusumawardhani N (2021)	Extracorporeal Membrane Oxygenation Used in Acute Respiratory Distress Syndrome with COVID-19: A Systematic Review and Meta-Analysis	Although the authors proposed a comparison of ECMO versus non-ECMO, the latter group in the SR considered patients who received high-flow nasal cannula therapy, non-invasive ventilation, mechanical ventilation or even none of these. In addition, they also included patients who did not have a diagnosis of ARDS.
Luo X (2020)	Supportive care for patient with respiratory diseases: an umbrella review	Document is an umbrella review of guidelines on supportive therapy for patients with respiratory diseases in general.
Nagraj S (2021)	Role of Invasive Mechanical Ventilation and ECMO in the Management of COVID-19: A Systematic Review	Most of the studies included in the SR were case reports or case series. In addition, the populations studied included patients without ARDS and, in some cases, were receiving added interventions such as convalescent plasma therapy.
De Oliveira TF (2020)	Extracorporeal Membrane Oxygenation in COVID-19 Treatment: A Systematic Literature Review	Most of the studies included in the SR corresponded to case reports or series, or observational studies without a control group. The remaining studies did not directly compare the use of ECMO versus mechanical ventilation, and there were even patients who received other therapies such as non-invasive ventilation. In addition, the populations analysed included patients without ARDS.
Ouyang L (2021)	Respiratory supports of COVID-19 patients in intensive care unit: A systematic review	The studies included in the SR did not directly compare the use of ECMO versus mechanical ventilation, with the exception of the study by Yang X et al., which is part of the evidence selected and analysed in our review.
Zhu Y (2020)	Extracorporeal membrane oxygenation versus mechanical ventilation alone in adults with severe acute respiratory distress syndrome: A systematic review and meta--analysis	Only one of the seven studies included in the SR compared ECMO versus mechanical ventilation in the ARDS population by COVID-19, which is part of the analysis in our review.
Suwalski P (2020)	Severe respiratory failure in the course of coronavirus disease 2019 treated with extracorporeal membrane oxygenation	Document was excluded because it corresponds to a case report with a systematic review of the literature (of case reports and case series).

Table S4. Summary of Recommendations of Each Clinical Practice Guideline

Clinical Practice Guideline	Recommendation/Statement
World Health Organization, 2021. "Living guidance for clinical management of COVID-19: Living guidance, 23 November 2021"	"In settings with access to expertise in ECMO, consider referral of patients who have refractory hypoxaemia (e.g., including a ratio of partial pressure of arterial oxygen [PaO_2] to the fraction of inspired oxygen [FiO_2] of <50 mmHg for 3 h, a PaO_2:FiO_2 of <80 mmHg for >6 h) despite lung protective ventilation" (this recommendation had a yellow symbol, which suggests that it is a "conditional recommendation in favour of an intervention or recommendation where special care is required in implementation").
Pan American Health Organization, 2021. "Guidelines for Care of Critically Ill Adult Patients with COVID-19 in the Americas. Version 3"	When possible, patients should receive extracorporeal membrane oxygenation (ECMO) or be referred to an ECMO centre in the following COVID-19 and ARDS critical cases: (i) patients under mechanical ventilation with refractory hypoxia who do not respond to recommended alternative therapies (ventilation optimization, use of rescue therapies, and mechanical ventilation in a prone position). The following patients should not use ECMO: (i) patients with terminal disease or central nervous system damage, or patients who do not want to be resuscitated or who refuse to use ECMO; (ii) patients with significant comorbidities; (iii) patients aged >65 years; and (iv) patients who have received mechanical ventilation for more than 7 days" (conditional recommendation, very low quality of evidence).
National Institutes of Health, 2022. "COVID-19 Treatment Guidelines Panel. Coronavirus Disease 2019 (COVID-19) Treatment Guidelines"	"There is insufficient evidence to recommend either for or against the use of extracorporeal membrane oxygenation (ECMO) in adults with COVID-19 and refractory hypoxemia".
Badulak J et al., 2021. "Extracorporeal Membrane Oxygenation for COVID-19: Updated 2021 Guidelines from the Extracorporeal Life Support Organization"	"(i) VV ECMO may be utilized for patients with COVID-19 and severe respiratory failure with expected outcomes comparable to patients supported with V-V ECMO pre-pandemic; and (ii) Rarely, children can require ECMO support for severe ARDS, myocarditis, or multisystem inflammatory disease in children; ECMO patient selection and management should follow conventional guidelines" (Evidence is not ranked and recommendations are not graded).
Alhazzani W et al., 2021. "Surviving Sepsis Campaign Guidelines on the Management of Adults With Coronavirus Disease 2019 (COVID-19) in the ICU: First Update"	"In mechanically ventilated adults with COVID-19 and refractory hypoxemia despite optimizing ventilation, use of rescue therapies, and prone positioning, we suggest using venovenous ECMO, if available, or referring the patient to an ECMO center" (weak recommendation, low quality of evidence).
Australian National COVID-19 Clinical Evidence Taskforce. "Australian guidelines for the clinical care of people with COVID-19, v51"	"(i) Consider early referral to an ECMO centre for patients developing refractory respiratory failure in mechanically ventilated adults with COVID-19 (despite optimising ventilation, including proning and neuromuscular blockers) (conditional recommendation, very low evidence certainty); (ii) Consider referral to an ECMO centre for venovenous ECMO in mechanically ventilated pregnant women with COVID-19 and refractory respiratory failure (despite optimising ventilation, including prone positioning). Delivery of the baby prior to ECMO to enhance maternal resuscitation should be considered on a case-by-case basis (consensus-based recommendation); and (iii) Consider early referral to an ECMO centre for venovenous or venoarterial ECMO in mechanically ventilated neonates, children and adolescents with COVID-19 with refractory respiratory or cardiovascular failure despite optimising other critical care interventions" (consensus-based recommendation).
Jin YH et al., 2020. "Chemoprophylaxis, diagnosis, treatments, and discharge management of COVID-19:	"ECMO is recommended to treat patients with critical COVID-19, and close monitoring of patient's vital signs is necessary during use. ECMO should be used in the following situations: (1) early stage (such as severe type with a course of less than 7 days) of critical patients with reversible condition; (2) severe hypoxemia:

An evidence-based clinical practice guideline (updated version)"	when using optimized PEEP, PaO_2/FiO_2 <100 mmHg after using neuromuscular blocker and prone ventilation; (3) excessive compensatory respiratory acidosis (pH < 7.15) when using optimized mechanical ventilation; (4) excessive inspiratory stress (plateau pressure >30 cmH_2O) when using lung protective ventilation; (5) using optimized mechanical ventilation setting, the mechanical power is ≥27 J/min; (6) using the optimized mechanical ventilation setting, there is right heart dysfunction due to acute pulmonary heart disease" (recommendation: Grade 1C: strong recommendation, low or very low quality evidence).
